# Tubulinopathy Presenting as Developmental and Epileptic Encephalopathy

**DOI:** 10.3390/children9081105

**Published:** 2022-07-23

**Authors:** Kun-Long Hung, Jyh-Feng Lu, Da-Jyun Su, Su-Jin Hsu, Lee-Chin Wang

**Affiliations:** 1 Department of Pediatrics, Fu-Jen Catholic University Hospital, New Taipei City 24352, Taiwan; aifighter@gmail.com; 2 Department of Pediatrics, Cathay General Hospital, Taipei City 106, Taiwan; hsj1002@hotmail.com.tw (S.-J.H.); leechinx@hotmail.com (L.-C.W.); 3 School of Medicine, Fu-Jen Catholic University, New Taipei City 24205, Taiwan; 049696@mail.fju.edu.tw; 4 Department of Pediatrics, National Taiwan University Hospital, Taipei City 100, Taiwan

**Keywords:** developmental and epileptic encephalopathy, pediatric, tubulinopathy, TUBA1A, TUBB2B

## Abstract

Tubulin proteins play a role in the cortical development. Mutations in the tubulin genes affect patients with brain malformations. The present report describes two cases of developmental and epileptic encephalopathy (DEE) due to tubulinopathy. Case 1, a 23-year-old boy, was found to have a brain malformation with moderate ventriculomegaly prenatally. Hypotonia was noted at birth. Seizures were noted on the 1st day with multifocal discharges on the EEGs, which became intractable to many anticonvulsants. Brain MRI showed marked dilated ventricles and pachy/polymicrogyri. He became a victim of DEE. A de novo mutation in TUBB2B was proven through next-generation sequencing (NGS). Case 2, a mature male baby, began to have myoclonic jerks of his limbs 4 h after birth. EEG showed focal sharp waves from central and temporal regions. Brain MRI showed lissencephaly, type I. The seizures were refractory initially. A de novo mutation in TUBA1A was proven at the 6th week through NGS. He showed the picture of DEE at 1 year and 2 months of age. The clinical features of the tubulinopathies include motor delay, intellectual disabilities, epilepsy, and other deficits. Our cases demonstrated the severe form of tubulinopathy due to major tubulin gene mutations. NGS makes the early identification of genetic etiology possible for clinical evaluation.

## 1. Introduction

Epileptic encephalopathy is a severe type of epilepsy that causes cognitive impairments due to frequent epileptic activities beyond what is expected from the underlying pathology [1,2]. Developmental encephalopathy is a condition of developmental impairment due to a nonprogressive brain condition without frequent epileptic activities. The term “developmental and epileptic encephalopathy (DEE)” used to be described as the coexistence of epileptic encephalopathy and developmental delay [3], but is now referred to as the condition of two comorbidities developing independently of each other, that is, developmental impairment related to both the underlying etiology and the epileptic encephalopathy, though they might share a common genetic etiology. The genetic etiologies responsible for DEE have been incrementally reported in recent decades, especially after the application of next generation sequencing (NGS) [3,4].

Tubulin proteins play a key role in the cortical development during neuronal proliferation, migration, differentiation and cortical lamination [5]. Mutations in the tubulin genes typically affect patients with complex malformations of cortical, commissural and posterior fossa and varying degrees of ventriculomegaly [6]. Tubulins are also the structural subunit protein to form the microtubules [7]. Mutations that cause dysfunction of the tubulin and microtubule-associated protein, known as tubulinopathy, can lead to a wide range of complex brain malformations [8]. The alpha and beta tubulins are the most common isoforms in humans that assemble the microtubules [9]. Since the first description in 2007 [10], at least eight genes encoding for α- (TUBA1A, TUBA8), β- (TUBB2A, TUBB2B, TUBB3, TUBB4A, TUBB) and γ-tubulins (TUBG1) have been reported clinically [11]. Mutations in the tubulin genes, highly expressed during CNS development, result in malformation of cortical development (MCDs). The present report describes two cases of DEE with malformation of the brain due to two distinctly different tubulinopathies.

## 2. Case Presentation

### 2.1. Case 1

#### Patient Information

**Figure 1:**
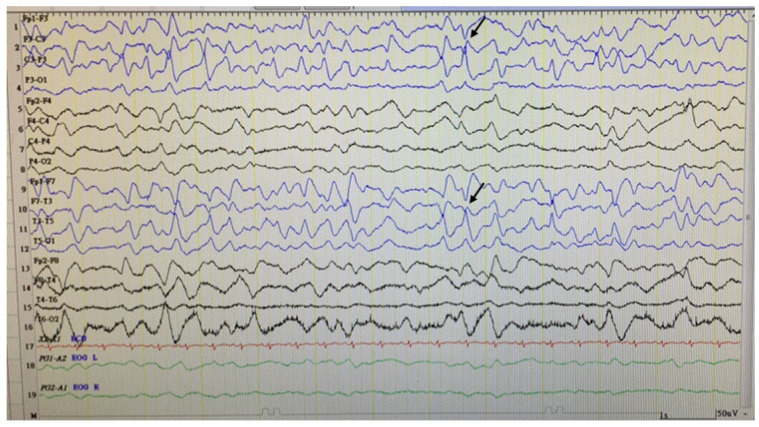
Sleep EEG of Case 1. It showed asymmetric slow background activities with focal discharges (arrows) from the left frontotemporal region.

**Figure 2:**
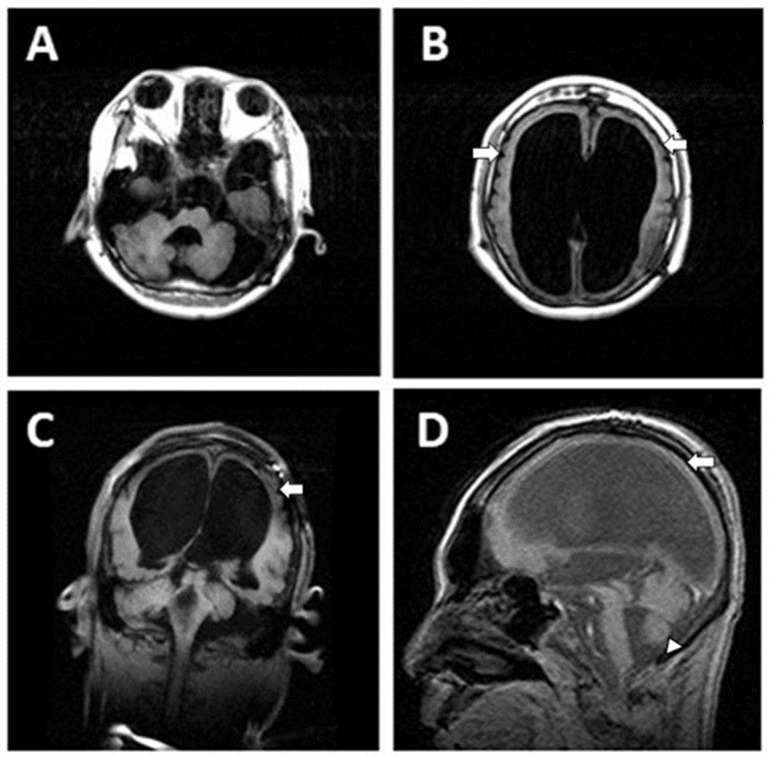
Brain MRI (T1WI) of Case 1 showed marked dilated ventricles with displaced cerebellum (**A**), dysgenesis of corpus callo-sum, pachy/polymicrogyria (white arrows in (**B**–**D**)) and small vermis (arrowhead in (**D**)).

**Figure 3:**
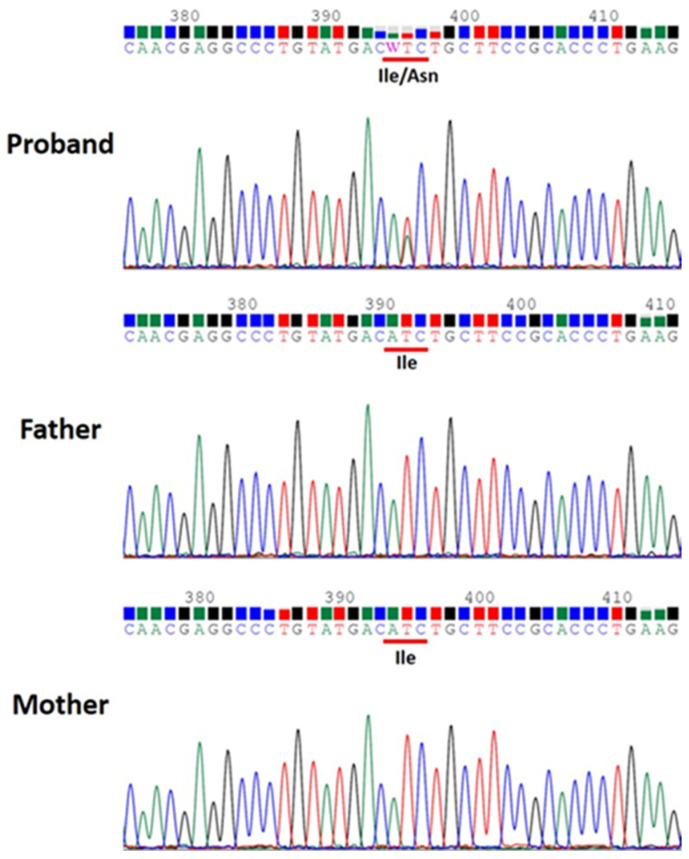
Sanger sequencing confirmed TUBB2B de novo mutation (NM_178012.5:c.629T>A; p.Ile210Asn) of Case 1 identified by whole exome sequencing (WES).

Case 1, a 23-year-old boy born to unrelated healthy parents, was found to have a brain malformation with moderate ventriculomegaly through prenatal ultrasonic checkups. The parents preferred to reserve the child. Hypotonia was noted at birth. Seizures were noted on the 1st day of life with multifocal discharges on the EEGs (Figure 1), which became intractable to many anticonvulsants including topiramate, lamotrigine and levetiracetam. Eventually, cannabiodiol was also tried. Brain MRI showed marked dilated ventricles with dysgenesis of corpus callosum, poor configuration of cortical gyri, and suspected pachy/polymicrogyri (Figure 2). He had other systemic problems including pseudo-obstruction of the lower GI tract (megacolon) and he underwent partial colectomy, colostomy, and gastrostomy at 2 years old. He became a victim of developmental and epileptic encephalopathy with severe motor and mental handicap. He could not walk and talk. He needed a lot of assistance with daily activities. A de novo mutation in TUBB2B (NM_178012.5: c.629T > A; p.Ile210Asn; dbSNP: rs1561826759) was later proven through whole exome sequencing (WES) via Illumina platform next-generation sequencing (NGS) and was confirmed by Sanger sequencing (Figure 3).

### 2.2. Case 2

#### Patient Information

**Figure 4:**
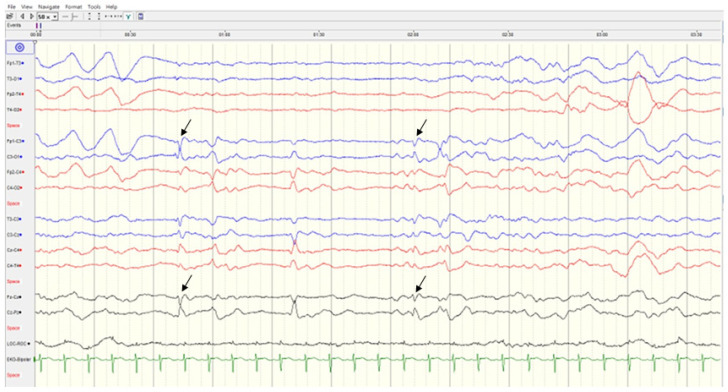
Sleep EEG of Case 2. It showed bilateral focal discharges (arrows) from central and temporal regions.

**Figure 5:**
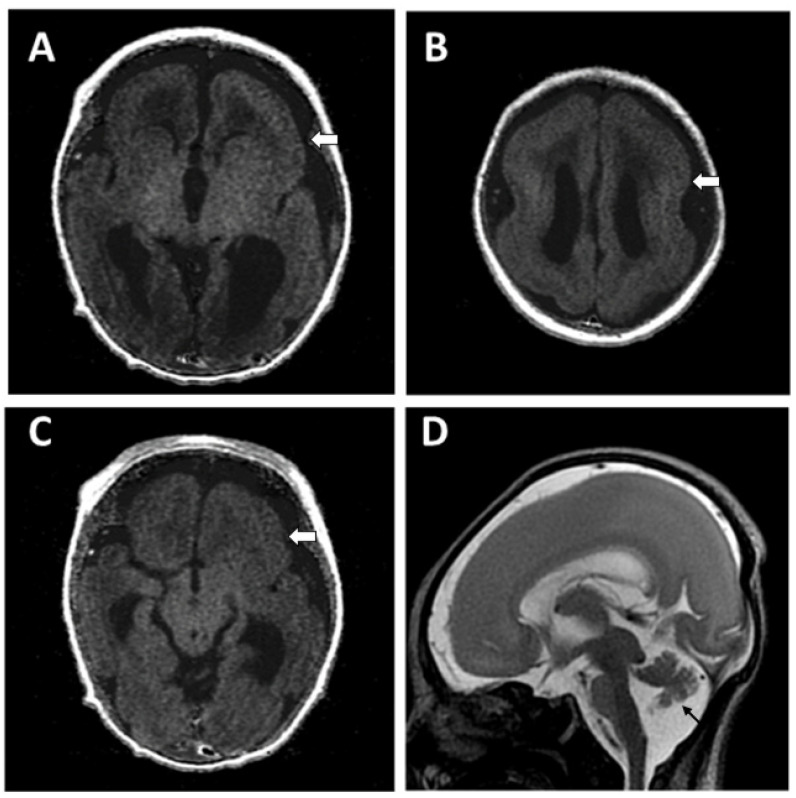
Brain MRI of Case 2 showed lissencephaly (white arrows), mildly dilated posterior horns of ventricles with dysgenesis of carpus callosum, and small vermis (arrow) ((**A**–**C**): T1WI, (**D**): T2WI).

**Figure 6:**
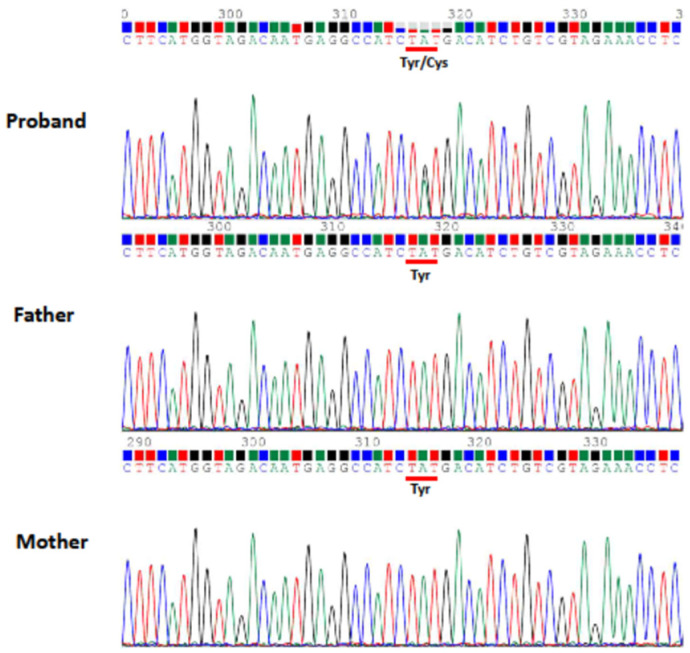
Sanger sequencing confirmed TUBA1A de novo mutation (NM_006009.4: c.629A>G; p.Tyr210Cys) of Case 2 identified by whole exome sequencing (WES).

Case 2, a mature male baby, born to unrelated healthy parents, began to have myoclonic jerks of his arms and legs 4 h after birth. Several episodes of apnea with cyanosis were also noted. EEG showed focal sharp waves from bilateral central and temporal regions (Figure 4). Brain CT showed the picture of lissencephaly, type 1. MRI confirmed the following findings: (1) Lissencepaly with shallow Sylvian fissures, compatible with classic lissencephaly, (2) dysgenesis of corpus callosum, (3) disproportional enlargement of ventricles and subarachnoid spaces, more on the left side, (4) presence of cavum septum pellucidum et vergae, and (5) flattened/hypoplastic anterior corpus callosum (Figure 5). The seizures became refractory to anticonvulsants with phenobarbital, valproic acid and levetiractam, and finally better controlled by lacosamide. He showed psychomotor delay. By the age of 1 year and 2 months, he could not crawl or walk. A de novo mutation in TUBA1A (NM_006009.4: c.629A>G; p.Tyr210Cys; dbSNP: rs1565627253) was proven at the 6th week of life through WES and was further confirmed by Sanger sequencing (Figure 6). The clinical presentations of Case 1 and Case 2 are summarized in Table 1.

## 3. Discussion

During the stages of neuronal migration and differentiation of cerebral cortical formation, the globular tubulins form heterodimers and then assemble into microtubules. Microtubules are cytoskeletal polymers that play a key role in the process of cortical development including neuronal proliferation, migration, and cortical lamination [5]. TUBA1A, TUBB2B and TUBB3 are responsible for more than 90% of known tubulin mutations associated with cortical malformations. Both TUBA1A and TUBB2B, in particular, have been associated with a wide spectra of cortical and extracortical malformations of the brain [9].

The recent advances in neuroimaging, especially, magnetic resonance (MR) imaging, have made it possible to correlate the phenotypic diagnosis of MCDs [12]. TUBA1A was the first reported tubulin gene mutation with abnormal cortical development [10]. From MR studies, TUBA1A mutations cause severe MCDs such as microlissencephaly, lissencephaly with pachygyria, and agyria, while TUBB2B-related tubulinopathies are characterized by focal or generalized polymicrogyria [13,14]. Other than the abovementioned cortical malformations, extracortical anomalies such as various degrees of asymmetric dysgenesis of the brainstem, distortion of the anterior part of the interhemispheric fissure, lack of Sylvian fissure operculation, and cerebellar anomalies can also be seen in MR images. Even prenatally, MR imaging can demonstrate severe forms of tubulinopathy, such as microlissencephaly, polymicrogyria, and kinked brainstem, and also mild forms such as asymmetric brainstem, callosal dysgenesis and commissural anomalies [15].

A recent cross-sectional quantitative analysis of the natural history of TUBA1A and TUBB2B tubulinopathies demonstrated striking information about these two mutation disease entities [11]. From their study cohorts, TUBA1A tubulinopathy is characterized by primary microcephaly, facial dysmorphisms, lissencephaly, and corpus callosal abnormalities, while TUBB2B tubulinopathy shows polymicrogyria and anomalies of basal ganglion and ventricle systems. Clinically, they became symptomatic in infancy at a mean age of 4 months (SD ± 5.3 months; N = 29) (TUBA1A group) and 6 months (SD ± 8.2 months; N = 17) (TUBB2B group), respectively, but show a big difference in diagnostic delay of 4.2 and 12.3 years, respectively, significantly higher in the TUBB2B group (*p* = 0.004). A generally milder course of TUBB2B compared with TUBA1A tubulinopathy might be the cause. It is not uncommon to find seizure occurrence during the neonatal stage for tubulinopathies [16]. In this report, our cases showed similar features. They shared common clinical features of tubulinopathies including motor delay, intellectual disabilities, various degrees of epilepsy, and other deficits. However, distinctive cortical pictures were mainly comparable, such as lissencephaly in TUBA1A and polymicrogyria in TUBB2B. Extracortical malformations were also obvious, such as dysgenesis of the corpus callosum and vermis hypoplasia in TUBA1A, and ventriculomegaly and asymmetry of the brainstem and cerebellum in TUBB2B tubulinopathy. For Case 1, although brain malformation was diagnosed prenatally, detailed genetic testing was not available; only in the last 10 years have serving hospitals been able to perform genetic testing with the WES. With this experience, we performed WES testing directly for Case 2 soon after the disease onset and got an accurate diagnosis. The progress of medical care is thus shown. The importance of the genetic approach to confirm the diagnosis from the onset of the disease to shorten the diagnostic delay is hereby stressed.

In silico predictions of the identified TUBB2B mutation (NM_178012.5:c.629T>A; NP_821080.1: p.Ile210Asn) revealed that it is probably damaging with a score of 0.999 (sensitivity: 0.09; specificity: 0.99) using PolyPhen-2 HumVar (http://genetics.bwh.harvard.edu/pph2/, accessed on 7 July 2022) and damaging with a score of 0 using SIFT4G (https://github.com/rvaser/sift4g, accessed on 7 July 2022). The Combined Annotation Dependent Depletion (CADD) PHRED score for this variant is 27.0, indicating that the nature of the variant is within the top 0.1% to 1% most deleterious. In silico predictions of the identified TUBA1A mutation (NM_006009.4: c.629A>G; NP_006000.2: p.Tyr210Cys) revealed that it is probably damaging with a score of 0.997 (sensitivity: 0.27; specificity: 0.98) using PolyPhen-2 HumVar (http://genetics.bwh.harvard.edu/pph2/, accessed on 7 July 2022) and damaging with a score of 0.009 using SIFT4G (https://github.com/rvaser/sift4g, accessed on 7 July 2022). The Combined Annotation Dependent Depletion (CADD) PHRED score for this variant is 27.0 indicating that the nature of the variant is within the top 0.1% to 1% most deleterious. The identified TUBB2B mutation (NM_178012.5:c.629T>A; NP_821080.1: p.Ile210Asn) was previously reported in ClinVar (VCV000624122.11) to be likely pathogenic, and in VarSome (https://varsome.com/, accessed on 7 July 2022) to be likely pathogenic according to The American College of Medical Genetics and Genomics (ACMG) classification guideline [17]. The identified TUBA1A mutation (NM_006009.4: c.629A>G; NP_006000.2: p.Tyr210Cys) was previously reported in ClinVar (VCV000625474.3) to be pathogenic and to occur de novo in a boy with lissencephaly [18], as well as in VarSome (https://varsome.com/, accessed on 7 July 2022) to be pathogenic according to the ACMG classification guidelines. Besides the common tubulin genes responsible for tubulin formation, there are also several microtubule-associated protein genes involved in cerebral formation. For example, LIS1, DCX, VLDLR, and RELN, are known to be associated with the LIS spectrum [13]. Most mutations in tubulin and microtubule-associated protein genes can manifest as microcephaly, cortical malformations, ventriculomegaly, and abnormalities of the corpus callosum, basal ganglion, brainstem and cerebellum, to various degrees. MR neuroimaging may provide the informative clue to understand the underlying tubilinopathies. The genetic approach is the diagnostic key to make a final conclusion on the disease entities. Among the genetic investigations, target panel testing and whole exome or whole genome sequencing based on the image findings are appropriate to explore the final etiology for these patients. In addition, identification of a gene mutation can help to deliver appropriate genetic counselling and care plans for the patients and family.

## Figures and Tables

**Table 1:** Clinical presentation of Case 1 (TUBB2B mutation) and Case 2 (TUBA1A mutation).

	Case 1	Case 2
Gender, age	Male, 23 years	Male, 1 years 2 months
Age of seizure onset	1 h	4 h
Consanguinity	no	no
Prenatal history	malformation of brain	unremarkable
Epilepsy	multifocal, refractory	focal to generalized, recurrent
Hypotonia	yes	yes
Developmental delay	global	global
Skeletal involvement	scoliosis	unknown
Feeding	gastrostomy	oral
Mutation	TUBB2B	TUBA1A

## Data Availability

All data are available upon request.
